# Effectiveness of negative pressure wound therapy for the wound of ileostomy closure: a multicenter, phase II randomized controlled trial

**DOI:** 10.1186/s12893-021-01446-2

**Published:** 2021-12-28

**Authors:** Koichiro Kojima, Mayu Goto, Yasuo Nagashima, Yoko Saito, Masaya Kawai, Shihori Takebe, Akiko Egawa, Mitsuko Tanba, Kazue Ishikawa, Hiroyoshi Matsuoka, Tadahiko Masaki, Eiji Sunami, Norihiko Ohura, Koji Teruya, Ken Eto, Keijiro Nozawa, Kazuhiro Sakamoto, Kimihiko Funahashi

**Affiliations:** 1 Department of Surgery, Kyorin University School of Medicine, 6-20-2, Shinkawa, Mitaka city, Tokyo, 181-8611 Japan; 2 Department of General and Gastroenterological Surgery, Toho University Omori Medical Center, Tokyo, Japan; 3 WOCN, Department of Nursing, Toho University Omori Medical Center, Tokyo, Japan; 4 Department of Coloproctological Surgery, Juntendo University School of Medicine, Tokyo, Japan; 5 WOCN, Department of Nursing, Juntendo University School of Medicine, Tokyo, Japan; 6 WOCN, Department of Nursing, The Jikei University School of Medicine, Tokyo, Japan; 7 WOCN, Department of Nursing, Kyorin University School of Medicine, Tokyo, Japan; 8 WOCN, Department of Nursing, Teikyo University School of Medicine, Tokyo, Japan; 9 Department of Paramedics, Kyorin University Faculty of Health Sciences, Tokyo, Japan; 10 Department of Plastic, Reconstructive and Aesthetic Surgery, Kyorin University School of Medicine, Tokyo, Japan; 11 Department of Health & Welfare, Kyorin University Faculty of Health Sciences, Tokyo, Japan; 12 Department of Surgery, The Jikei University School of Medicine, Tokyo, Japan; 13 Department of Surgery, Teikyo University School of Medicine, Tokyo, Japan

**Keywords:** Negative pressure wound therapy, Ileostomy closure, Wound healing, Surgical site infection, Purse-string suture, Randomized controlled trial, Phase II trial

## Abstract

**Background:**

The American Society of Surgery and American Society for Surgical Infections issued guidelines for surgical site infections (SSIs) in December 2016. These guidelines recommend a purse-string suture (PSS) for stoma closure as it facilitates granulation and enables open wound drainage. This study investigated the effect of using negative pressure wound therapy (NPWT) along with standard PSS and aimed to determine the optimal period of NPWT use.

**Methods:**

The patients were divided into three groups as follows: Group A, postoperative wound management alone with gauze exchange as the representative of conventional PSS; Group B, the performed management was similar to that of Group A plus NPWT for 1 week; and Group C, the performed management was similar to that of Group A plus NPWT for 2 weeks. Regarding objective measures, the wound reduction rate was the primary outcome, and the incidence of SSIs, length of hospital stay, and wound healing duration were the secondary outcomes.

**Results:**

In total, 30 patients (male: 18, female: 12) were enrolled. The average age was 63 (range: 43–84) years. The wound reduction rate was significantly higher in Group B than in Group A on postoperative days (PODs) 7 (66.1 vs. 48.4%, p = 0.049) and 10 (78.6 vs. 58.2%, p = 0.011), whereas no significant difference was observed on POD 14. Compared with Group A, Group C (POD 7: 65.9%, POD 10: 69.2%) showed an increase in the wound reduction rate on POD 7, although the difference was not significant (p = 0.075). SSIs were observed in Groups B (n = 2) and C (n = 2) (20%) but not in Group A (0%).

**Conclusions:**

The most effective duration of NPWT use for ileostomy closure with PSS in terms of the maximum wound reduction rate was from PODs 3 to 10. However, NPWT did not shorten the wound healing duration. NPWT may reduce the wound size but should be used with precautions for SSIs. The small sample size (30 cases), the use of only one type of NPWT system, and the fact that wound assessment was subjective and not blinded were the limitations of this study. Further studies are needed to confirm our findings.

*Trial registration:* UMIN Clinical Trials Registry; UMIN000032174 (10/04/2018).

## Background

In recent years, the widespread adoption of laparoscopic and robot-assisted surgeries and development of advanced surgical techniques and devices have enabled lower anastomosis during rectal cancer surgery. Although anal preservation has become possible, a temporary stoma is often constructed to prevent anastomotic sepsis in many cases, such as when the anastomosis is close to the anus. A temporary stoma that is constructed to prevent the complications of colorectal surgery is closed 3–6 months after the initial surgery. The complication rate can be as high as 20–40%, with the most common complication being surgical site infections (SSIs), as the wound of stoma closure is at a site where the feces was expelled [1–3].

Since 1995, purse-string suture (PSS) for stoma closure has been reported to be useful for preventing SSIs, mainly in Europe and the United States. PSS for stoma closure is a method of suturing the dermis in an annular shape to create a semi-open wound, that significantly reduces the incidence of SSIs compared with the conventional simple wound closure [4, 5]. The incidence rate of SSI with this method was reported to be 0–5% [6]. Recently, guidelines in Europe and the United States recommended PSS as a method of wound closure following stoma creation [7]. In Japan, PSS is the standard treatment in several institutions; however, it requires approximately 1 month for complete wound healing. Therefore, the wound of stoma closure with PSS is considered to be an intractable wound. There are some problems associated with wound management post-PSS, including the need for regular wound cleaning and replacement of gauze, as well as the cost of treatment tools. Furthermore, patients are often discharged from the hospital before achieving complete wound healing; hence, monitoring the wound and expenditure related to the medical materials needed for its management become the patients’ responsibility.

By shrinking the wound edges, increasing blood flow, promoting granulation, reducing edema, and removing excess exudates, negative pressure wound therapy (NPWT) promotes wound healing through the application of negative pressure to wounds, such as pressure ulcers, acute traumatic wounds, chronic intractable wounds, and postoperative wound infections [8–11]. It has been widely used in Europe and the United States since 1995 and has been covered by the medical insurance of the Ministry of Health, Labor, and Welfare of Japan since 2010. Recently, the efficacy of incisional NPWT (iNPWT) for the prevention of wound complications in closed surgical incisions has been reported [12]. However, in this study, the wound was not completely closed but partially opened. Additionally, NPWT was used to promote granulations of the subcutaneous tissue, which is considered different from iNPWT. Therefore, the combined use of NPWT with stoma closure is expected to facilitate wound healing by increasing wound shrinkage, reducing the wound healing time and SSI incidence, and reducing the personal and economic burden on medical staff, patients, and caretakers.

Several case reports and retrospective studies evaluating the effectiveness of NPWT for stoma closure have reported a shorter wound healing duration and lower incidence of SSIs using NPWT [13–16]. Azuma et al. reported that NPWT for stoma closure significantly reduced the average wound volume from 2.3 mL on the day of surgery to 0.16 mL on postoperative day (POD) 7 in 20 cases. Naito et al. reported that the incidence rate of wound infection was 6.7% and the average wound healing duration was 18 days in 30 cases. Moreover, compared with previous reports on PSS without NPWT, the incidence rate of wound infection was expected to decrease, while the wound healing duration was expected to shorten. Obeid et al. reported that NPWT for stoma closure reduced the frequency of visits to the dressing clinic, suggesting a significant implication for cost savings [15]. There is only one reported of a prospective single-center randomized controlled trial [17]; however, it did not report a significant effect on the complete wound healing duration and incidence of SSIs. Additionally, there are no reports of multicenter studies. The effectiveness of combined NPWT with stoma closure has not yet been established. In addition, factors, such as the size of foam, time of insertion, and the duration of dressing, are unclear. Therefore, in this prospective, multicenter randomized controlled trial, we aimed to explore the effectiveness and optimal duration of NPWT use for stoma closure.

## Methods

### Patients

This prospective randomized controlled trial was conducted between March 2018 and March 2019 at five university hospitals in Tokyo, Japan. We enrolled patients with an ileostomy who were scheduled for closure surgery and obtained their written consent for participation in the study after explaining them the purpose of the study using a form approved by the ethics committee of each institution. The exclusion criteria were as follows: the presence of comorbidities that were strongly suspected to affect wound healing, such as familial adenomatous polyposis or inflammatory bowel disease; hemodialysis; poorly controlled diabetes mellitus (glycated hemoglobin level of ≥ 7.5%); steroid medications; and difficulty in changing dressings due to dementia, psychiatric illness, or postoperative delirium. The patients were randomly assigned to the treatment groups post-enrollment by the central allocation method. For the random assignment, every case was simply randomized at an allocation ratio of 1:1:1, and the random sequences were generated using a random number table associated with the statistical accountability of this study. The trial was not interrupted, and enrollment was terminated when the expected number of patients was reached.

### Surgical techniques (PSS)

**Fig. 1:**
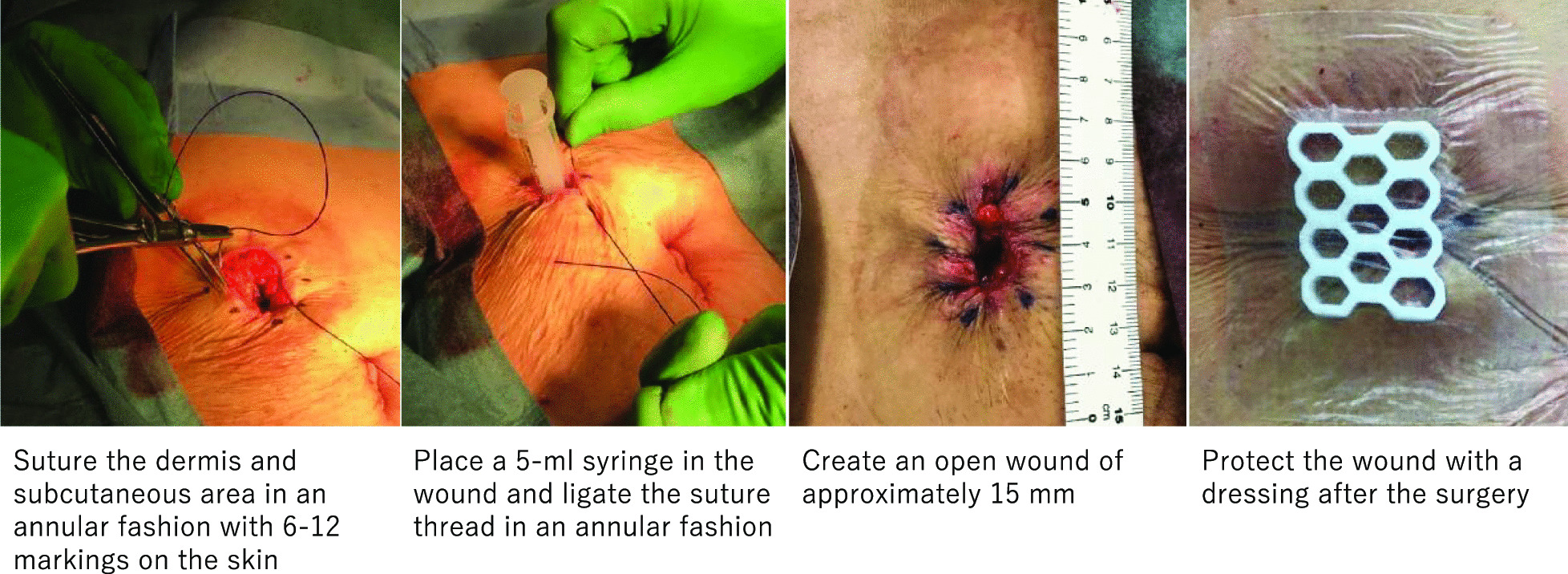
Purse-string suture. The performed method of PSS is presented. We used 1 PDS Plus as the suture and a 5-mL syringe was used to create an open wound. *PSS* purse-string suture

After closure of the peritoneum and rectus abdominis sheath during the stoma closure surgery, the dermis and subcutaneous tissue were sutured in a ring with 6–12 sutures using a monofilament absorbent suture (1 PDS Plus, CTB 40 mm 1/2 Circle; Ethicon Inc., Somerville, NJ, USA). Then, it was ligated with a 5-mL syringe, creating an open wound with a diameter of approximately 15 mm for drainage. After surgery, the wound was protected with a dressing sheath that was removed the following day (Fig. 1).

### Wound management

#### Group A (control group)

The dressing was removed on POD 1, and the wound was washed daily thereafter. Moreover, the gauze was mainly used and changed repeatedly until epithelialization occurred.

#### Group B (NPWT weekly-use group)

**Fig. 2:**
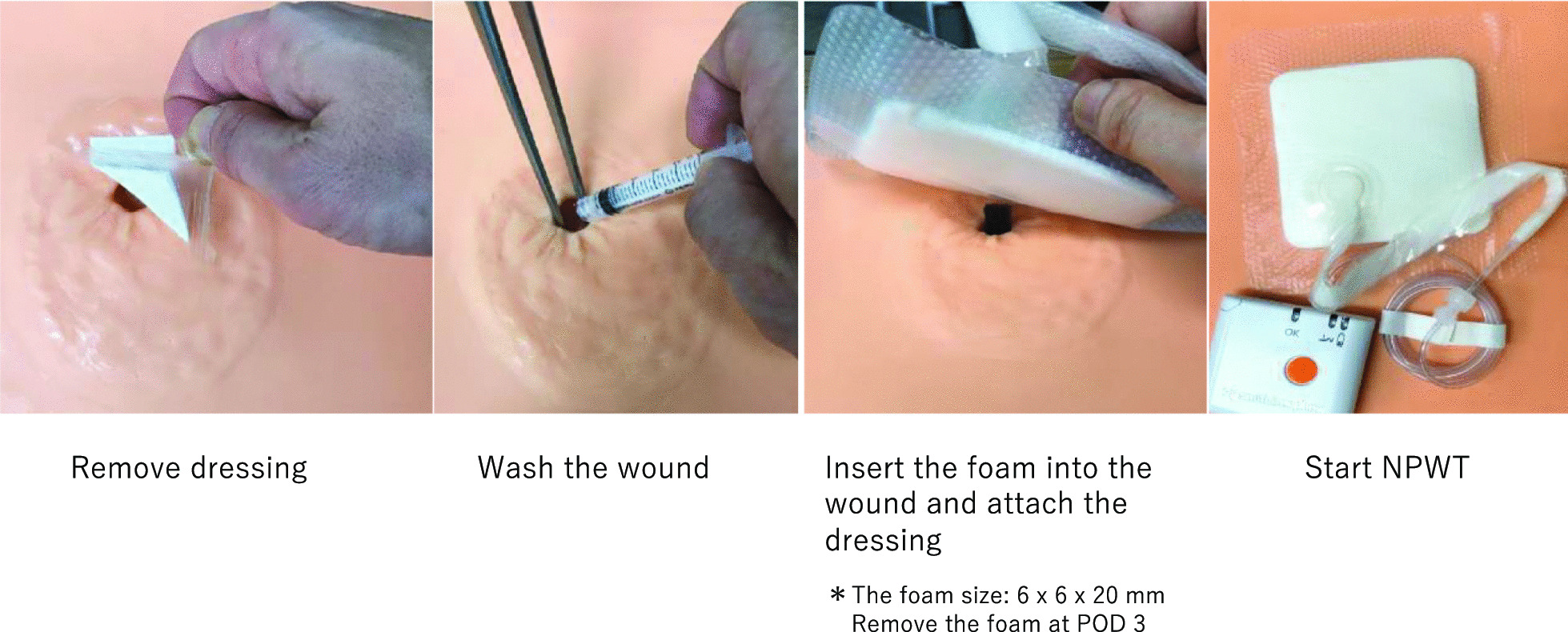
Wound treatment in Groups B and C. The wound management method using the PICO system for PSS is presented. The foam inserted when applying PICO was removed on POD 3. *PSS* purse-string suture, *POD* postoperative day

The day after surgery, the dressing was removed, and a foam insert measuring 6 × 6 × 20 mm was introduced into the wound after wound cleaning. The foam was cut from Smith & Nephew Foam Filler (Smith & Nephew Co. Ltd., London, United Kingdom). Subsequently, NPWT (PICO Single Use Negative Pressure Wound Therapy System; Smith & Nephew Co. Ltd.), which operates at a negative pressure of 80 mmHg, was initiated. On POD 3, the dressing and foam were removed. After wound cleaning, NPWT was continued without inserting a foam piece into the wound. NPWT was completed on POD 7, and appropriate wound cleaning was performed. The gauze was changed until epithelialization occurred, similar to the procedure performed in the control group (Fig. 2).

#### Group C (NPWT fortnightly use group)

The day after surgery, the dressing was removed, and a similar foam to that used in Group B was introduced into the wound after wound cleaning. Subsequently, NPWT, which operates at a negative pressure of 80 mmHg, was initiated. On POD 3, the dressing and foam were removed. After wound cleaning, NPWT was continued without inserting a foam piece into the wound. On PODs 7 and 10, the dressings were removed, wound cleaning was performed, and NPWT was continued. NPWT was completed on POD 14, and appropriate wound cleaning was performed. The gauze was changed until epithelialization occurred, similar to the procedure in the control group; however, if exudates were not observed to adhere to the dressing while changing it on PODs 7 and 10, these were considered the dates of wound healing (Fig. 2).

### Endpoint criteria

**Fig. 3:**
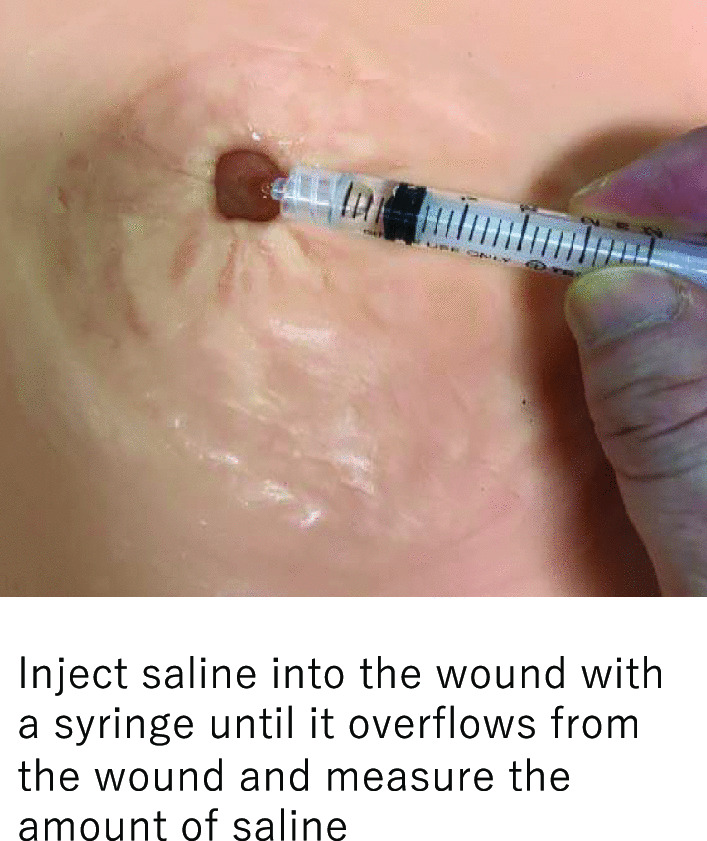
Measurement of wound volume. The wound volume was measured by injecting saline solution into the wound using a 1-mL syringe

The primary endpoint was selected as the rate of wound reduction from the original wound volume. The incidence of SSI, wound size (long diameter × short diameter, depth), healing time, and complication rate (excluding SSI) were also evaluated. The wound volume was measured on PODs 1, 3, and 7 and compared with that on POD 1 to calculate the reduction rate. To measure the wound volume, a saline solution was injected into the wound, and the volume was calculated based on the amount of fluid retained (Fig. 3). Wound healing was defined as the absence of exudates adhering to the gauze or skin protector applied to the wound. SSI was diagnosed if pus was observed in the wound. SSIs were classified as superficial incisional, deep incisional, and organ/space SSIs, depending on the site of infection.

### Statistical analyses

Continuous data are presented as medians and ranges. Comparative analysis of continuous variables was performed using the Mann–Whitney U test. The test for three independent sample data is non-parametric; therefore, the Kruskal–Wallis test was used for this analysis. Categorical variables were compared using the chi-square and Fisher’s exact tests. The level of significance was set at p < 0.05.

**Fig. 4:**
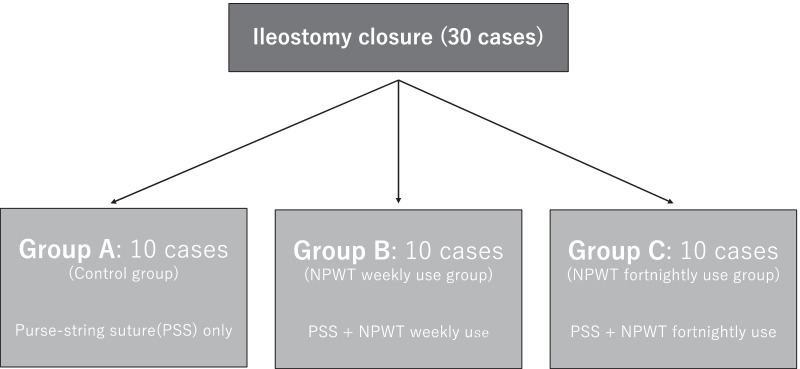
Study flowchart. The 30 patients enrolled were divided into the groups

**Fig. 5:**
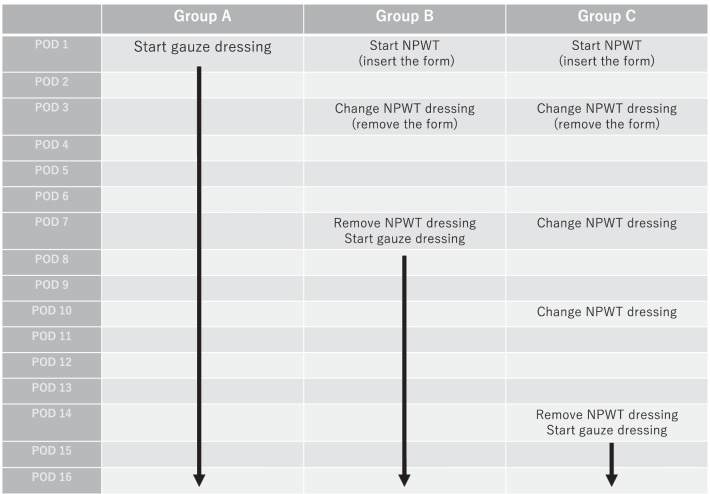
Study protocol. The treatment schedule for each of the three groups

Before conducting the study, an acceptable sample size was determined; however, there have been no studies examining the reduction rate of stoma closure wounds with PSS, and because we were unable to determine the number of participants, we were forced to conduct an exploratory study. Therefore, there is no clear basis for setting the number of registered cases. Given that our study was designed to investigate the efficacy and safety of NPWT in an exploratory manner, the number of enrolled cases was set at 30 (10 cases for each intervention group; Figs. 4 and 5).

## Results

**Fig. 6:**
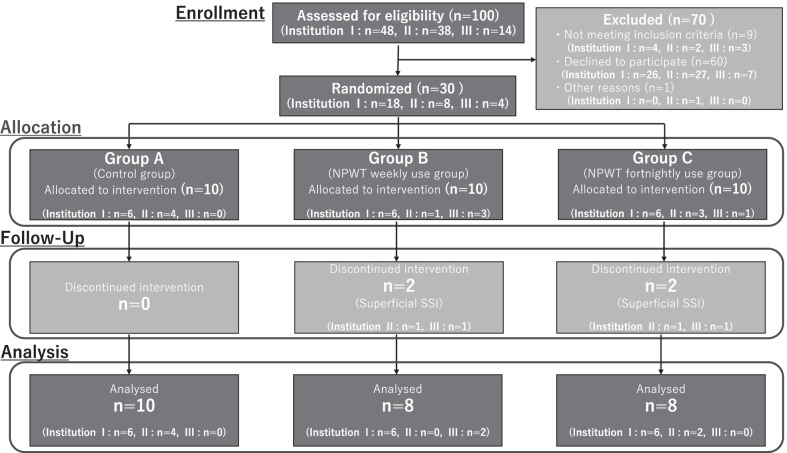
CONSORT flow chart. The process, by which patients assigned to each group, was analyzed

Five university hospitals agreed to participate in this study. However, the approval of the ethics committee of each institution was not obtained until study initiation; therefore, the study was conducted only at three institutions. Thirty patients from three university hospitals were enrolled in the study; there were no statistical differences in demographic and clinical characteristics between the groups (Table 1). All the patients enrolled in this study had an American Society of Anesthesiologists class I or II classification; none of them had undergone preoperative chemotherapy or radiotherapy, and none was diagnosed with renal failure. Preoperative diseases that may have affected the outcome were hypertension in eight cases (Group A: three, Group B: three, Group C: two), hypercholesterolemia in three (Group A: one, Group B: zero, Group C: two), ischemic heart disease in one (Group A: zero, Group B: one, Group C: zero), and cerebral infarction in one (Group A: zero, Group B: zero, Group C: one). No significant difference was observed in the baseline characteristics among Groups A, B, and C. The CONSORT flow chart is presented in Fig. 6, and the postoperative complications are shown in Table 2. The overall incidence rate of wound infection in the cohort was 13.3% (4/30 cases: 0, 2, and 2 cases in Groups A, B, and C, respectively). All SSIs were superficial SSIs.

**Table 1:** Patients’ demographic and clinical characteristics

Median [range]	Group A	Group B	Group C	p-value
Institutions, I:II:III Sex, M:F	6:4:0 6:4	6:1:3 8:2	6:3:1 4:6	0.248
Age	64 [48–82]	69 [48–75]	70 [43–84]	0.846
Height (cm)	162.5 [148.0–181.0]	165.0 [153.0–176.1]	157.7 [138.0–175.0]	0.325
Weight (kg)	59.2 [36.0–84.6]	59.6 [46.4–69.8]	48.8 [39.8–73.9]	0.161
BMI (kg/m^2^)	22.2 [16.4–30.1]	21.8 [16.5–23.8]	20.3 [17.0–26.2]	0.394
Thickness of subcutaneous fat (cm)	1.7 [0.3–2.8]	2.1 [0.9–3.2]	2.0 [0.8–3.8]	0.606
Area of the ileostomy opening (cm^2^)	9.8 [6.3–13.5]	11.7 [7.3–25.0]	7.5 [3.4–31.5]	0.224
Height of the ileostomy stoma (cm)	2.0 [1.0–3.0]	1.5 [1.0–3.5]	2.0 [1.3–2.2]	0.284

Institution I: Toho University Omori Medical Center, II: Juntendo University Hospital, III: Kyorin University Hospital. POD postoperative day

**Table 2:** Postoperative complications

	Group A	Group B	Group C	p-value
Superficial incisional SSI, n(%)	0 (0)	2 (20)	2 (20)	0.507

SSI surgical site infection

**Fig. 7:**
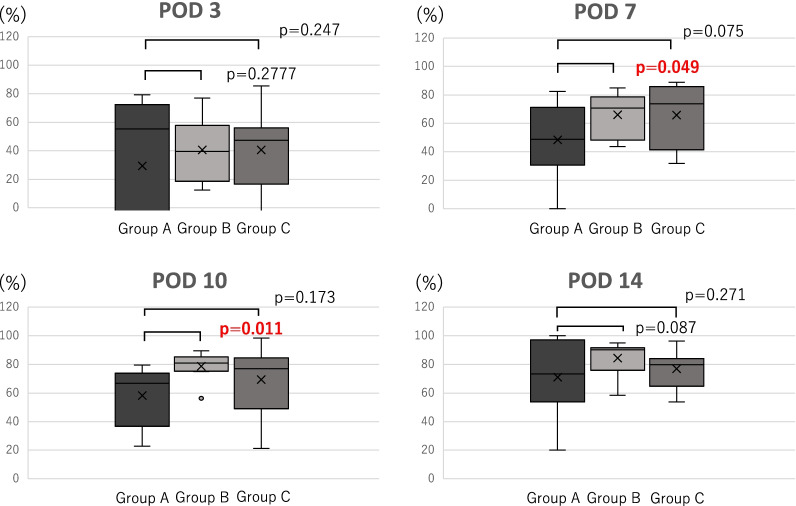
Reduction rate. The comparison of the wound reduction rate in the postoperative course of each group is presented. There was a significant difference in the reduction rate between Groups A and C at PODs 7 and 10

Given that the patients with SSIs (n = 4) were not suited for comparison with those without SSIs, they were excluded from the other study endpoints. On POD 3, the average wound reduction rate was 32.3% (29.4%, 40.5%, and 40.7% in Groups A, B, and C, respectively), and no significant difference was found between Group A (control) and Groups B and C (Group A vs B: p = 0.278, Group A vs C: p = 0.284). On POD 7, the average wound reduction rate was 31.4% (Group A: 48.4%, Group B: 66.1%, Group C: 65.9%). Compared with Group A, Group B showed a significant increase in the wound reduction rate (p = 0.049). There was no significant difference in the wound reduction rate between Groups A and C. Although Group C demonstrated an increase in the wound reduction rate, the difference was not significant (p = 0.075). On POD 10, the average wound reduction rate was 67.2% (58.2%, 78.6%, and 69.2% in Groups A, B, and C, respectively), and compared with Group A, Group B showed a significant increase in the wound reduction rate (p = 0.011); while there was no significant difference between Groups A and C (p = 0.173). On POD 14, the average wound reduction rate was 72.5% (71.0%, 84.4%, and 76.9% in Group A, B, and C, respectively), and no significant difference was found between the groups (Group A vs. B, p = 0.087; Group A vs. C, p = 0.271) (Fig. 7). In addition, there were no significant differences in the wound reduction rates between institutions in most situations (Table 3).

**Table 3:** Average wound reduction rate for each institution

(%)	POD 3	POD 7	POD 10	POD 14
A: Institution I	36.1	56.1	64.0	77.9
II	19.2	36.8	46.7	60.7
III	–	–	–	–
p-value	0.318	0.182	0.194	0.206
B: Institution I	44.4	71.2	77.9	87.5
II	–	–	–	–
III	28.9	50.9	80.6	74.9
p-value	0.105	0.030	0.359	0.295
C: Institution I	32.9	58.6	69.0	77.6
II	74.0	87.8	69.8	53.8
III	–	–	–	–
p-value	0.039	0.010	0.490	0.157

Institution I: Toho University Omori Medical Center, II: Juntendo University Hospital, III: Kyorin University Hospital. POD postoperative day

**Fig. 8:**
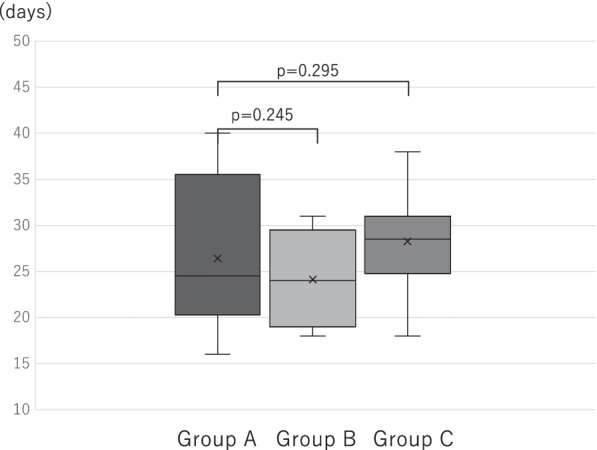
Wound healing period. The comparison of the healing time between the groups is presented. No significant differences were observed

The average wound healing duration was 26.7 days (Group A: 26.4 days, Group B: 23.1 days, Group C: 30.6 days), and no significant difference was found among the three groups (Group A vs. B, p = 0.2448; Group A vs. C, p = 0.2946) (Fig. 8). In addition, common complications in gastrointestinal surgery such as postoperative bleeding and adhesive bowel obstruction, were evaluated; interestingly, no adverse events other than SSIs occurred in patients undergoing NPWT (Groups B and C). The average length of postoperative hospital stay was 16.0 days (Group A: 15.6 days, Group B: 16.6 days, Group C: 15.5 days). No significant difference was observed among the Groups A, B, and C (Group A vs. B, p = 0.3968; Group A vs. C, p = 0.6888; Group B vs. C, p = 0.3774). NPWT was completed during hospitalization in all patients, and no complications occurred in the outpatient setting.

## Discussion

In this study, the wound reduction rate did not differ between the groups on POD 3, whereas it was superior in Group B compared with that in Group A on PODs 7 and 10; no difference was detected on POD 3 possibly because of NPWT use during the inflammatory phase of the wound healing process, which could not provide the expected effects because of the presence of inflammation. In contrast, NPWT use after the end of the inflammatory phase provided notable benefits, including rapid drainage of exudates, promotion of angiogenesis, proliferation of tissue, and reduction of physical wound volume by negative pressure, resulting in effective wound reduction on PODs 7 and 10 [18, 19]. Although no significant difference was observed between Groups A and C, the wound reduction rate of Group C was similar to that of Group B. Given that Groups B and C were managed in the same manner until POD 7, the absence of a significant difference between the two groups might be attributed to the small number of cases. Although there was an increase in the wound reduction rate on PODs 7 and 10, the wound healing duration did not differ between the groups, suggesting that NPWT is most effective during the proliferative phase of the wound healing process. Furthermore, early recovery from the inflammatory phase and rapid transition to the proliferative and remodeling phases may have a positive impact on the integrity of the wound during healing.

Although the incidence of SSIs following stoma closure is high (2–41%) [1, 20], PSS, the wound closure method employed to prevent infection, has been reported to greatly reduce this incidence to 0–5%. Therefore, the incidence of SSIs is not a suitable primary endpoint for phase II studies. Several retrospective reports have suggested that the combination of PSS and NPWT may lead to a shorter wound healing time [16]; however, no difference was found in a previous randomized controlled trial [17]. It is difficult to prove the benefit of NPWT in terms of wound healing time; therefore, the primary endpoint in this study was the wound reduction rate. Bleeding from the wound may cause obstruction of the NPWT dressings and worsen the drainage of exudate; therefore, the dressing was applied the day after surgery, after confirming the absence of bleeding. Although the dressings for the NPWT used in this study were designed to be used for 7 days, they were changed in a shorter period of time as it was necessary to monitor for complications such as bleeding and infection.

In this study, the incidence rate was as high as 13.3%, despite the exclusion of high-risk cases. PSS reduces the rate of infection by opening and draining the wound. Given that an open wound takes time to heal, NPWT is used to promote granulation and to shorten the wound healing duration [10]. Conversely, there has been a long-standing concern that closing the wound might lead to the development of infections. In this study, SSIs only occurred in Groups B and C that underwent NPWT, suggesting that SSIs are associated with closed dressings. We used the PICO Single Use Negative Pressure Wound Therapy System because the procedure is relatively simple and can be used in an outpatient setting; however, infections occurred only in patients who underwent NPWT, which is plausibly explained by the inability of the multi-layered dressing to absorb viscous exudates. If NPWT had been performed with a higher pressure and direct foam aspiration, such as with the RENASYS System (Smith & Nephew Co. Ltd.) or the ActiV.A.C. Therapy System (3 M, Saint Paul, MN, USA) the patient may have been successfully treated without infection. These NPWT types are common for open wounds; they are different from the PICO system used in this study. This potential difference needs to be investigated in future studies.

We found that the wound infections occurred only in certain institutions, indicating that the cause of SSIs in the NPWT group may have been attributed to unfamiliarity of the staff at some institutions, and that the assessment of wound infections may not have been standardized in each institution. Moreover, there may have been a risk of detection bias because the assessment of wounds was not structured, and the treatment received was not blinded. The importance of cautiously using NPWT for wound infections was reconfirmed, and further validation is required after the standardization of wound dressing procedures and wound assessment.

Postoperative complications, all of which were superficial SSIs, were observed in four of the 20 patients who underwent NPWT. Although delayed wound healing was observed, wound closure was observed in all patients without prolongation of hospital stay; thus, the safety of the treatment was proven in this study. We observed a significant difference in the wound reduction rates on PODs 7 and 10. The PICO dressings require replacement every 7 days, suggesting that the optimal duration of NPWT use is from PODs 3 to 10; thus, these days may thus represent the most effective duration of NPWT use for wound reduction.

This study had some limitations, which include its small sample size (30 cases); absence of certain preoperative and perioperative characteristics, such as smoking habits; use of only one type of NPWT system; and the fact that wound assessment was subjective and not blinded. This study reported a significant difference in the wound reduction rate between Groups A and B on POD 7; therefore, the mean difference and standard deviation between the two groups were calculated from these data (mean difference between the two groups: 15.6, standard deviation: 23.6). The sample size, which was calculated using a one-tailed test with a significance level of 0.05 and power of 0.8, was 29 in each group. The power, which was calculated using a one-tailed test with a sample size of 10 cases in each group according to the mean difference and standard deviation between the two groups, was 0.43. Although the sample size in this study was not sufficient, it was considered to be within the acceptable range.

The results of this study were uncertain due to the aforementioned limitations; however, there have been no similar multicenter, prospective studies, conducted to date. Therefore, this study may serve as a stepping stone toward evaluating the role of NPWT for the wound of ileostomy closure.

## Conclusion

The most effective duration of NPWT use for ileostomy closure with PSS in terms of the maximum wound reduction rate was from POD 3 to 10. Although NPWT reduced the wound size, it did not shorten the wound healing duration. Additionally, the procedure should be performed with appropriate infection control measures, such as occasional removal of the dressings to monitor the wound. Further studies are needed to confirm our findings.

## Data Availability

The datasets used and/or analyzed during the current study are available from the corresponding author on reasonable request.

## Abbreviations

NPWT: Negative pressure wound therapy
POD: Postoperative day
PSS: Purse-string suture
SSI: Surgical site infections
